# Improving Quality of Cause-of-Death Reporting in New York City

**DOI:** 10.5888/pcd10.130227

**Published:** 2013-07-18

**Authors:** Ann Madsen, Elizabeth Begier

**Affiliations:** Author Affiliation: Elizabeth Begier, New York City Department of Health and Mental Hygiene, New York, New York.

In “Survey of New York City Resident Physicians on Cause-of-Death Reporting, 2010,” (Survey) (1), Wexelman et al reported on New York City (NYC) resident physicians’ self-reported beliefs and behaviors relating to NYC’s cause-of-death reporting system. In mid-2010, they asked residents about their experiences in the prior 1 to 3 years (June 2007–June 2010); they found that only one-third of residents believed cause-of-death reporting was accurate, and about half had knowingly reported at least one inaccurate cause of death, often at the request of a medical examiner or admitting staff or because “the system” would not accept the correct cause. Residents reported most frequently using heart disease as an inaccurate cause of death. These findings reflect previous US-wide (2) and NYC (3) reports of cardiovascular disease death overreporting and poor quality cause-of-death reporting. Survey authors recommended that the system “allow reporting of more causes” and that resident physicians receive better training but did not describe successful quality-improvement efforts undertaken since these residents’ experiences 3 to 6 years ago.

In 2008, NYC’s health department created a first-in-the-nation eLearning instructional course on cause-of-death reporting and demonstrated that the course taught medical residents to accurately report cause of death (4). In January 2010, NYC mandated this eLearning course for Electronic Death Registration System (EDRS) users. According to the Survey, only 21.5% of resident respondents reported completing the course by May 2010. Enforcing the requirement has proved challenging and labor-intensive. NYC plans to automate the enforcement process in its next EDRS, currently under development, by requiring completion of the eLearning course before user credentialing. NYC’s eLearning course was recently updated to improve clarity and provide additional case examples (5). Furthermore, NYC disseminated 2 City Health Information Bulletins on cause-of-death reporting in 2008 and 2012 and made guidance on cause-of-death-reporting available as a pocket card and as a poster, and via the New York City Department of Health and Mental Hygiene website (5).

In June 2009, NYC initiated 2 intervention efforts targeting hospitals with poor cause-of-death reporting. The first targeted 8 hospitals, 7 of which were teaching hospitals, that had excessive reporting of cardiovascular disease as cause of death (6). The health department conducted conference calls and in-service trainings to educate administrators about their hospitals’ poor cause-of death reporting and to train physicians and hospital staff on how and why to properly document cause of death. We emphasized reporting underlying cause of death, the cause that initially set off the chain of events leading to death, rather than only the intermediate or most proximal causes. The intervention significantly improved these hospitals’ reporting (6) and enhanced public health statistics (7). During initial conference calls and in-service trainings, we learned that physicians had been misinterpreting the automated data checks that had been implemented with the EDRS in 2008 to improve cause-of-death reporting. One data check restricted reporting of nonspecific causes of death, including septic shock, without an additional explanatory condition. Septic shock, an intermediate cause of death, generally is caused by another condition (eg, urinary tract infection) or risk factor (eg, immune suppression). Such underlying causes are important to public health statistics and disease prevention. As reported both in the Survey and to us verbally during our outreach, physicians misinterpreted the data check to mean that septic shock was not “accepted.” EDRS was altered to allow physicians to override these messages if indicated.

During 2011 and 2012, we conducted another similar intervention at an additional 12 NYC hospitals with similar successful results. In 2012, we partnered with the Survey’s authors to provide training at St. Luke’s–Roosevelt hospitals on proper cause-of-death reporting practices. Finally, we have coordinated with the NYC’s medical examiner’s office to harmonize cause-of-death messaging and reduce any inadvertent negative effect of the cause-of-death screening that the medical examiner’s office performs for all cremations.

In summary, we found that improving the quality of cause-of-death data requires a close examination of potential barriers to accurate cause-of-death reporting within death registration processes (both internal and external to EDRS) as well as clinician and hospital staff training.
